# Evaluation of Large Language Models for Radiologists’ Support in Multidisciplinary Breast Cancer Teams: Comparative Study

**DOI:** 10.2196/68182

**Published:** 2026-02-02

**Authors:** Hong Jiang, Chun Yang, Wenbin Zhou, Cheng-liang Yin, Shan Zhou, Rui He, Guanghui Ran, Wujie Wang, Meixian Wu, Juan Yu

**Affiliations:** 1 Faculty of Medicine Macau University of Science and Technology Macao China; 2 Department of Statistics Zhuhai Clinical Medical College of Jinan University Zhuhai China; 3 Qiandongnan Prefecture Hospital of Traditional Chinese Medicine Kai Li China; 4 Guangdong Provincial Key Laboratory of Tumor Interventional Diagnosis and Treatment Zhuhai China; 5 Department of Medical Innovation Research Chinese PLA General Hospital Beijing China; 6 Cancer Virology Program UPMC Hillman Cancer Center University of Pittsburgh School of Medicine Pittsburgh, PA United States; 7 Department of Microbiology and Molecular Genetics University of Pittsburgh School of Medicine Pittsburgh, PA United States; 8 Grammar and Cognition Lab (GraC) Department of Translation & Language Sciences Universitat Pompeu Fabra Barcelona Spain; 9 Department of Radiology Shenzhen Second People's Hospital The First Affiliated Hospital of Shenzhen University Shenzhen China

**Keywords:** breast cancer, large language models, LLMs, radiology assistance, clinical decision-making, ACR BI-RADS, NCCN guidelines, radiologist, National Comprehensive Cancer Network, American College of Radiology Breast Imaging-Reporting and Data System

## Abstract

**Background:**

Artificial intelligence tools, particularly large language models (LLMs), have shown considerable potential across various domains. However, their performance in the diagnosis and treatment of breast cancer remains unknown.

**Objective:**

This study aimed to evaluate the performance of LLMs in supporting radiologists within multidisciplinary breast cancer teams, with a focus on their roles in facilitating informed clinical decisions and enhancing patient care.

**Methods:**

A set of 50 questions covering radiological and breast cancer guidelines was developed to assess breast cancer. These questions were posed to 9 popular LLMs and clinical physicians, with the expectation of receiving direct “Yes” or “No” answers along with supporting analysis. The performances of the 9 models, including ChatGPT-4.0, ChatGPT-4o, ChatGPT-4o mini, Claude 3 Opus, Claude 3.5 Sonnet, Gemini 1.5 Pro, Tongyi Qianwen 2.5, ChatGLM, and Ernie Bot 3.5, were evaluated against that of radiologists with varying experience levels (resident physicians, fellow physicians, and attending physicians). Responses were assessed for accuracy, confidence, and consistency based on alignment with the 2024 National Comprehensive Cancer Network Breast Cancer Guidelines and the 2013 American College of Radiology Breast Imaging-Reporting and Data System recommendations.

**Results:**

Claude 3 Opus and ChatGPT-4 achieved the highest confidence scores of 2.78 and 2.74, respectively, while ChatGPT-4o led in accuracy with a score of 2.92. In terms of response consistency, Claude 3 Opus and Claude 3.5 Sonnet led the pack with scores of 3.0, closely followed by ChatGPT-4o, Gemini 1.5 Pro, and ChatGPT-4o mini, all recording impressive scores exceeding 2.9. ChatGPT-4o mini excelled in clinical diagnostics with a top score of 3.0 among all LLMs, and this score was also higher than all physician groups; however, no statistically significant differences were observed between it and any physician group (all *P*>.05). ChatGPT-4 also had a higher score than the physician groups but showed comparable statistical performance to them (*P*>.05). Across radiological diagnostics, clinical diagnosis, and overall performance, ChatGPT-4o mini and the Claude models achieved higher mean scores than all physician groups. However, these differences were statistically significant only when compared to fellow physicians (*P*<.05). However, ChatGLM and Ernie Bot 3.5 underperformed across diagnostic areas, with lower scores than all physician groups but no statistically significant differences (all *P*>.05). Among physician groups, attending physicians and resident physicians exhibited comparable high scores in radiological diagnostic performance, whereas fellow physicians scored somewhat lower, though the difference was not statistically significant (*P*>.05).

**Conclusions:**

LLMs such as ChatGPT-4o and Claude 3 Opus showed potential in supporting multidisciplinary teams for breast cancer diagnostics and therapy. However, they cannot fully replicate the intricate decision-making processes honed through clinical experience, particularly in complex cases. This highlights the need for ongoing artificial intelligence refinement to ensure robust clinical applicability.

## Introduction

Breast cancer is one of the most common malignancies in women worldwide, with over 2 million diagnoses each year, and it remains the leading cause of cancer-related deaths in women [1,2]. According to a World Health Organization departmental news report in 2021, breast cancer mortality and incidence rates are higher in low- and middle-income countries than in high-income nations, largely due to disparities in early detection and treatment. However, the biological characteristics of breast cancer vary significantly, including imaging features, pathological traits, and lymph node assessment, making early screening and personalized treatment challenging yet crucial.

A mature early screening system relies on well-established professional teams and standardized, specialized guidelines. Although there have been recent advancements, developing countries still face significant challenges in breast cancer care in terms of multidisciplinary coordination among specialist teams (surgeons, oncologists, pathologists, and radiologists), well-balanced medical resources, and adequately trained physicians [3,4]. Furthermore, many physicians rely on empiricism and practice medicine rather than evidence-based guidelines [5,6]. Therefore, there is an urgent need for effective artificial intelligence (AI) tools to support health care professionals in resource-limited settings, improve diagnostic accuracy, and enhance patient outcomes [7,8].

In recent years, several prominent large language models (LLMs) have emerged, including ChatGPT (OpenAI), Gemini Advanced (Google LLC), Claude (Anthropic), Tongyi Qianwen (Alibaba Corporation), ChatGLM (Zhipu AI), and Ernie Bot (Baidu). These models, trained on extensive datasets, offer significant potential in health care by generating complex text through deep learning. ChatGPT, for instance, gained rapid popularity, attracting over 1 million users within days of its release. (knowledge base updated as of October 2023). Studies have shown its potential in medicine, performing well on the United States Medical Licensing Examination and the Board of Radiology-style examination [9,10]. ChatGPT can help radiologists simplify cumbersome diagnostic imaging descriptions [11,12]. Notably, the recommendations provided by ChatGPT for the clinical management of early-diagnosed breast cancer closely align with decisions made by multidisciplinary teams (MDTs) [13]. ChatGPT has indicated a level of congruence with the National Comprehensive Cancer Network (NCCN) guidelines in identifying a wide range of therapeutic agents for the treatment of advanced metastases or advanced primary tumors [14]. However, recent studies highlighted significant limitations in ChatGPT-4.0, revealing a “curse reversal,” where a model trained on “A is B” may struggle to infer “B is A” [15].

The rapid advancement of AI has ushered in a new era of innovation, with the emergence and continuous refinement of various LLMs profoundly transforming the health care sector. This study evaluates the performance of various LLMs in addressing breast cancer guideline-related questions, comparing them with radiologists of varying expertise levels to assess their potential in enhancing radiologists’ diagnostic capabilities.

## Methods

### Study Design

**Figure 1:**
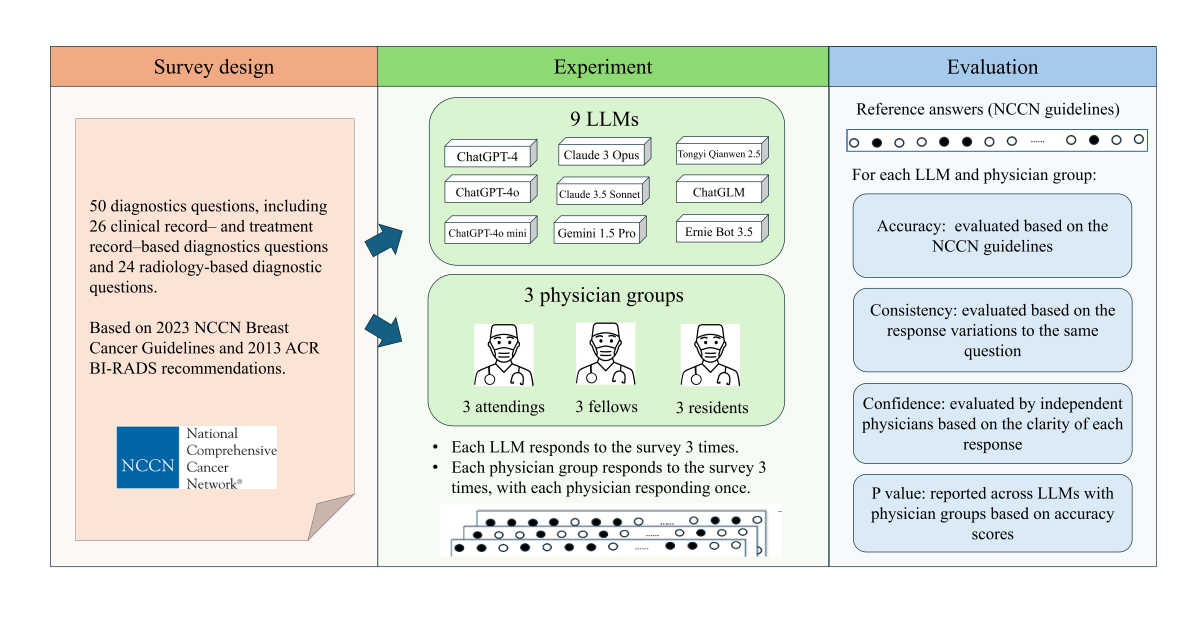
Graph in the full-text abstract: the study design, including question categorization, experiment, and evaluation workflow. ACR BI-RADS: American College of Radiology Breast Imaging-Reporting and Data System; LLM: Large Language Model; NCCN: National Comprehensive Cancer Network.

The study aimed to evaluate the performance of 9 LLMs in breast cancer imaging diagnosis and to compare their performance with that of breast imaging specialists. We developed a set of 50 questions focused on breast cancer diagnosis and treatment based on the 2013 version of the American College of Radiology Breast Imaging-Reporting and Data System (ACR BI-RADS) classification and the 2024 NCCN Breast Cancer Guidelines. The first 24 questions focused on radiological diagnostics, while the remaining 26 questions pertained to clinical diagnosis and treatment. Questions were then presented to breast radiologists and the 9 LLMs for diagnostic responses. The accuracy, confidence, and consistency of each model in responding to the diagnostic queries were assessed. Furthermore, we conducted a comparative analysis of each model’s performance against radiologists possessing varying levels of clinical experience. The study design is provided in Figure 1.

### Participants and Models

The 9 LLMs included ChatGPT-4.0, ChatGPT-4o, ChatGPT-4o mini, Claude 3 Opus, Claude 3.5 Sonnet, Gemini 1.5 Pro, Tongyi Qianwen 2.5, ChatGLM, and Ernie Bot 3.5. In addition, the study recruited a total of nine board-certified radiologists, stratified into three distinct experience levels: (1) resident physicians (n=3; 1-5 years of postlicensure experience), undergoing subspecialty training to develop foundational clinical expertise; (2) fellow physicians (n=3; 5-10 years of experience), having completed residency and pursuing advanced fellowship training (1-3 years) to specialize in breast imaging diagnostics; and (3) attending physicians (n=3; >10 years of experience), senior specialists with independent diagnostic authority, responsible for clinical decision-making and trainee supervision. This hierarchical cohort enabled comparative benchmarking of LLM performance against progressive stages of radiologist expertise. Each physician received the same set of 50 questions.

### Questions Design

The set of 50 questions was developed based on guidelines extracted from the 2024 NCCN Breast Cancer Guidelines and the 2013 ACR BI-RADS diagnostic criteria, which are widely regarded as the gold standards in clinical breast cancer diagnosis. These criteria are commonly adhered to by physicians in clinical breast cancer diagnosis. In this study, we used these same standards to evaluate the responses provided by the LLMs and physicians to ensure validity and fair comparison. The questions covered a range of common clinical diagnostic decision-making scenarios, providing radiological evidence, such as mammography, ultrasound, and magnetic resonance imaging, as well as clinical information indicators such as medical history, physical examination findings, and laboratory test results, across preoperative, intraoperative, and postoperative stages.

### Procedure

Nine physicians were divided into 3 groups based on their experience levels, with each physician answering all 50 questions once. They were prohibited from consulting guidelines or reference materials and were required to complete the questions within 30 minutes. Each physician independently completed the assessment to minimize recall bias. The study aimed to simulate real-world conditions by controlling time and eliminating external aids.

All LLMs were queried via the Google application programming interface on a single date (August 21, 2024) without any additional context (such as task background or medical knowledge priming) and generated responses accordingly. Each LLM independently answered every question 3 times. Standard responses were defined as “yes” or “no.” A response was classified as “yes” if it demonstrated a strong affirmative inclination, supported by compelling theoretical evidence and logical reasoning. Conversely, a response was classified as “no” if it showed a clear negative inclination, backed by equally robust theoretical evidence and sound logic. If an LLM produced an ambiguous response, lacked sufficient evidence, or contained flawed reasoning, it was prompted to reanswer. If the reanswered response remained unsatisfactory, 2 senior physicians with over 10 years of experience were recruited to classify the response based on their professional judgment, thereby evaluating the AI model’s accuracy. In cases of disagreement, the evaluators reevaluated the response based on the 2024 NCCN Guidelines and the 2013 ACR BI-RADS criteria to reach a consensus through discussion.

All LLM-generated responses were anonymized, randomized, and then assessed by evaluators who were blinded to the source (LLM identity) of each response. The accuracy of each response was assessed against the diagnostic NCCN guidelines. Responses consistent with the guidelines were assigned a score of 1, while inconsistent responses received a score of 0. The accuracy score for each question was calculated by summing the scores of the 3 responses. The overall accuracy performance of both the LLMs and physician groups was determined by averaging the accuracy scores across the 50 questions, resulting in a score ranging from 0 to 3. Subsequently, the accuracy performance of the LLMs was compared to that of the physician groups through rigorous statistical analysis.

### Confidence and Consistency

Confidence refers to the clarity of responses, categorized as either “confident” or “nonconfident.” A confident answer provides an explicit “yes” or “no” to questions, while a nonconfident answer lacks a direct “yes or no” statement, despite potentially containing detailed explanations, requiring additional prompting to elicit a definitive response. Confident answers received are scored 1, while nonconfident answers are scored 0. The confidence score for each question is calculated by summing the scores from 3 independent response attempts. Each LLM’s overall confidence performance is assessed by averaging these scores across 50 questions, resulting in a score ranging from 0 to 3. The confidence ratio represents each LLM’s average confidence score divided by the maximum possible score (ie, 3).

Consistency evaluates whether an LLM provides uniform responses across 3 independent attempts to answer the same question. If all 3 answers are identical, the responses are considered consistent, regardless of their alignment with guideline criteria. For consistency assessment, an LLM receives 3 points if all 3 answers to a question are identical, and 2 points if one answer differs from the others. Each LLM’s consistency performance is evaluated by averaging these scores across 50 questions, with possible scores ranging from 2 to 3. The consistency ratio is defined as each LLM’s average consistency score divided by the maximum possible score (ie, 3).

### Statistical Analysis

**Figure 2:**
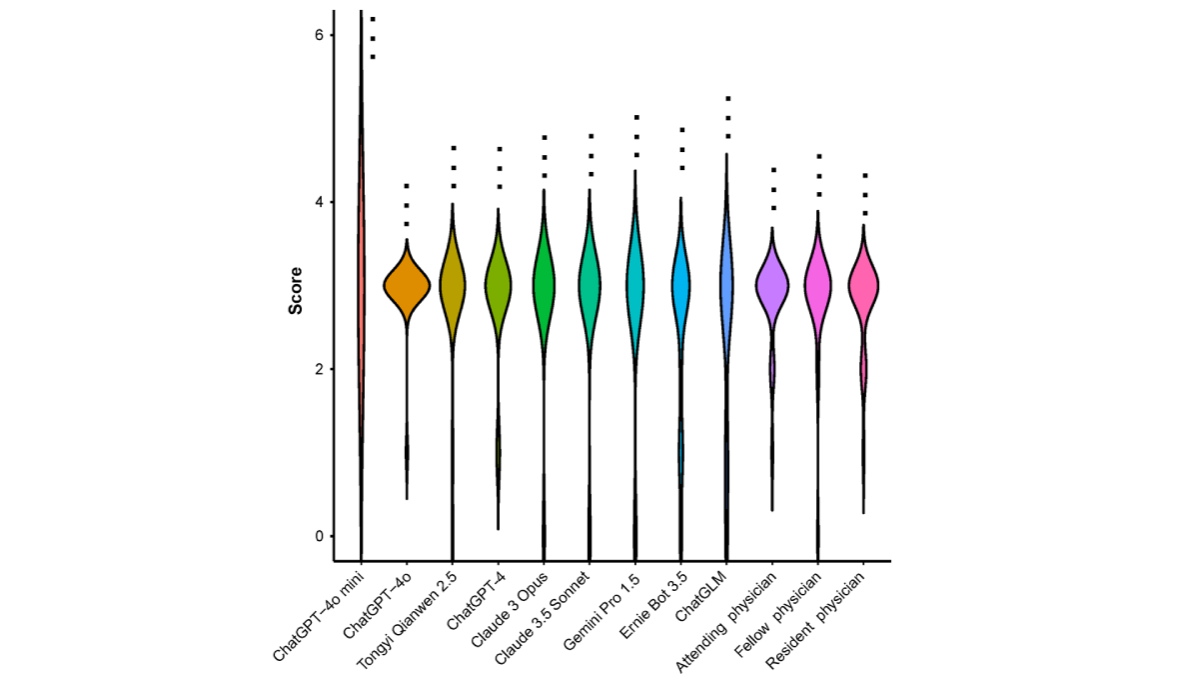
Accuracy scores of large language models (LLMs) compared to physicians in clinical diagnosis and treatment questions of breast cancer. 3-star or dot symbols on each violin from top to bottom represent *P* values compared to each LLM to attending, fellow, and resident physicians, using the Wilcoxon rank-sum test. Symbols are as follows: “***” represents *P*<.001, “**” represents .001≤*P*<.01, “*” represents .01≤*P*<.05, and “black dots” represents P≥.05.

**Figure 3:**
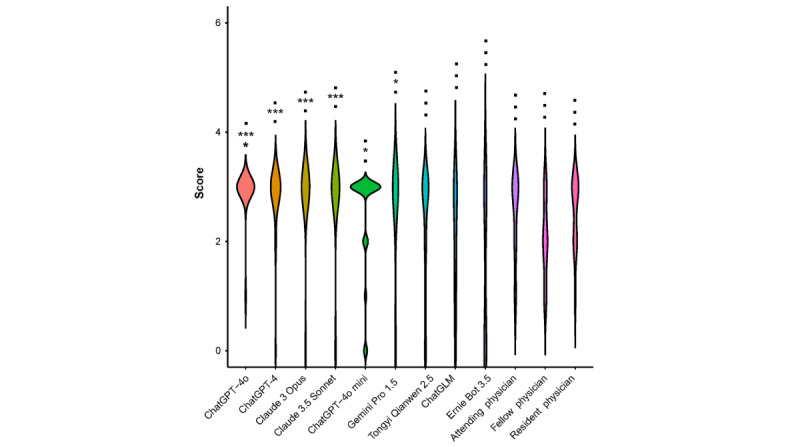
Accuracy scores of large language models (LLMs) compared to those of physicians in radiological diagnosis questions of breast cancer.3-star or dot symbols on each violin from top to bottom represent *P* values compared to each LLM to attending, fellow, and resident physicians, using the Wilcoxon rank-sum test. Symbols are as follows: “***” represents *P*<.001, “**” represents .001≤*P*<.01, “*” represents .01≤*P*<.05, and “black dots” represents P≥.05.

**Figure 4:**
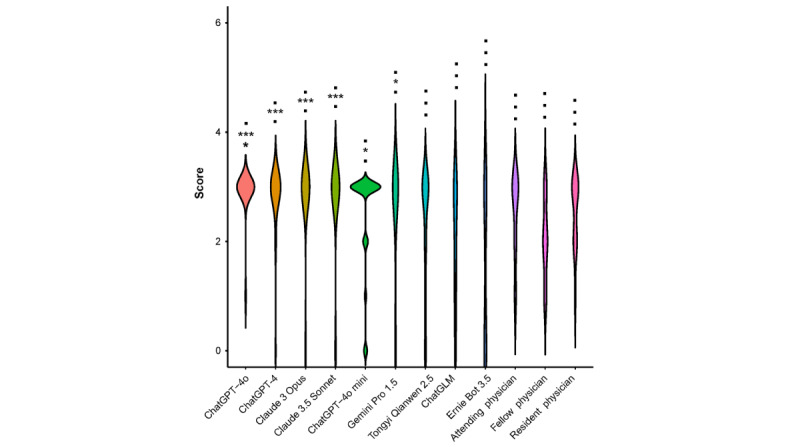
Accuracy scores of large language models (LLMs) compared to those of physicians in totalfor all 50 questions of breast cancer questions.3-star or dot symbols on each violin from top to bottom represent *P* values compared to each LLM to attending, fellow, and resident physicians, using the Wilcoxon rank-sum test. Symbols are as follows: “***” represents *P*<.001, “**” represents .001≤*P*<.01, “*” represents .01≤*P*<.05, and “black dots” represents P≥.05.

R software (version 4.4.2; R Foundation for Statistical Computing) was used for data management and statistical analysis. Continuous variables were described using means, and specific group instances were expressed as absolute values. The Wilcoxon rank-sum test was used for score comparisons between 2 different groups. We opted not to perform a Kruskal-Wallis test (for overall multigroup comparison) before pairwise analyses because our research focus was on hypothesis-driven, targeted comparisons between 2 specific groups: each LLM vs each physician subgroup (attendings, fellows, and residents). This design directly aligned with our aim—to evaluate whether single LLMs differ in performance from radiologists at distinct experience levels—rather than testing for overall differences across all groups. Differences between groups were considered significant at *P*<.05. *P* value correction for multiple comparisons was applied using the Benjamini-Hochberg method. Adjusted *P* values are provided in Multimedia Appendix 1, Tables 2-4, and corresponding Figures 2-4 to present direct pairwise comparison results; raw *P* values for multiple comparisons are provided exclusively in Multimedia Appendix 2.

### Ethical Considerations

Upon review by the Ethics Committee of Zhuhai People’s Hospital, the information and content provided by the researcher did not involve the collection, storage, or use of human samples, and, therefore, met the requirements for exemption from ethical review.

## Results

### Representative Examples

Textbox 1 provides a representative selection of question-answer examples from the 9 LLMs, while a more comprehensive collection of cases is provided in the Multimedia Appendix 3.

**Representative examples of questions and answers from the 9 large language models (LLMs).**

**Sample question:**

- Can an oval, hypoechoic lesion, growing nonparallel or vertically, with a heterogeneous echo pattern and obscured margins on breast ultrasound, be classified as Breast Imaging Reporting and Data System (BI-RADS 4b)?

**ChatGPT-4 response:**

- Yes, an oval, hypoechoic lesion growing nonparallel (vertically) with a heterogeneous echo pattern and obscured margins observed on breast ultrasound could be classified as BI-RADS 4b. This classification indicates a moderate suspicion of malignancy, meaning the lesion exhibits characteristics that are more suspicious than those typically found in BI-RADS 4a but not as definitively malignant as those in BI-RADS 4c.
- The characteristics you described—nonparallel orientation (often termed “taller than wide”), heterogeneous echo pattern, and obscured margins—suggest a higher level of concern. These features are often associated with a greater likelihood of malignancy:
  - Nonparallel or vertical orientation: this orientation is more typical of malignant lesions, as benign masses often grow in parallel with the skin.
  - Heterogeneous echo pattern: this can indicate variability within the lesion, which is more common in malignant tumors than in benign growths.
  - Obscured margins: this suggests that the lesion’s borders are not clearly defined, which can be an indication of invasive growth, a characteristic more typical of malignant processes.
- Conclusion: a lesion under 5 mm without a lipid core showing a washout pattern on magnetic resonance imaging should be approached with a high degree of suspicion for malignancy. This situation requires proactive management, including possible biopsy and close imaging follow-up, to ensure timely diagnosis and treatment.

**ChatGPT-4o response:**

- Yes, an oval, hypoechoic lesion that is growing nonparallel (vertically oriented), with a heterogeneous echo pattern and obscured margins on breast ultrasound can be classified as BI-RADS 4b. BI-RADS 4b is used to describe lesions with a moderate suspicion of malignancy.
- Key features of BI-RADS 4b:
  - Oval shape: while an oval shape can suggest a benign lesion, other features, such as orientation, margin appearance, and internal echo pattern, significantly influence the level of suspicion.
  - Nonparallel growth (vertically oriented): a lesion that grows nonparallel, or “taller than wide,” raises concern for malignancy. This orientation is often associated with invasive tumors because malignant lesions tend to invade tissue in a vertical direction.
  - Heterogeneous echo pattern: a heterogeneous echo pattern, where the lesion appears uneven or varied in echogenicity, is suspicious because it can indicate the presence of both solid and cystic components, or areas of differing cellular density, which is common in malignant tumors.
  - Obscured margins: obscured or indistinct margins are another red flag for malignancy. Clear, well-defined margins are typically seen in benign lesions, while indistinct margins suggest invasion into surrounding tissues.
- In conclusion, the combination of nonparallel growth, heterogeneous echo pattern, and obscured margins justifies a BI-RADS 4b classification, meaning the lesion has a moderate risk of malignancy and requires biopsy for a definitive diagnosis.

**ChatGPT-4o mini response:**

- Yes, an oval, hypoechoic lesion with a nonparallel (vertically oriented) growth pattern, heterogeneous echo pattern, and obscured margins on breast ultrasound can be classified as BI-RADS 4b.
- Here’s why this classification might be appropriate:
  - Oval shape: while typically less suspicious than irregular shapes, an oval shape alone does not rule out malignancy.
  - Hypoechoic: a darker appearance on ultrasound is often associated with both benign and malignant lesions.
  - Nonparallel growth pattern: a vertical or irregular growth pattern can be more concerning, as it may suggest infiltration into surrounding tissues.
  - Heterogeneous echo pattern: variability in echo patterns within a lesion can be indicative of more complex internal structures, which may be suggestive of malignancy.
  - Obscured margins: margins that are not well-defined or are irregularly shaped can be a sign of malignancy.
- BI-RADS 4b indicates a moderate suspicion of malignancy, with a likelihood of 10%-50%. This classification suggests that while there is a notable suspicion of cancer, the probability is not as high as in BI-RADS 4c. A biopsy is recommended to obtain a definitive diagnosis.

### Confidence Level

**Figure 5:**
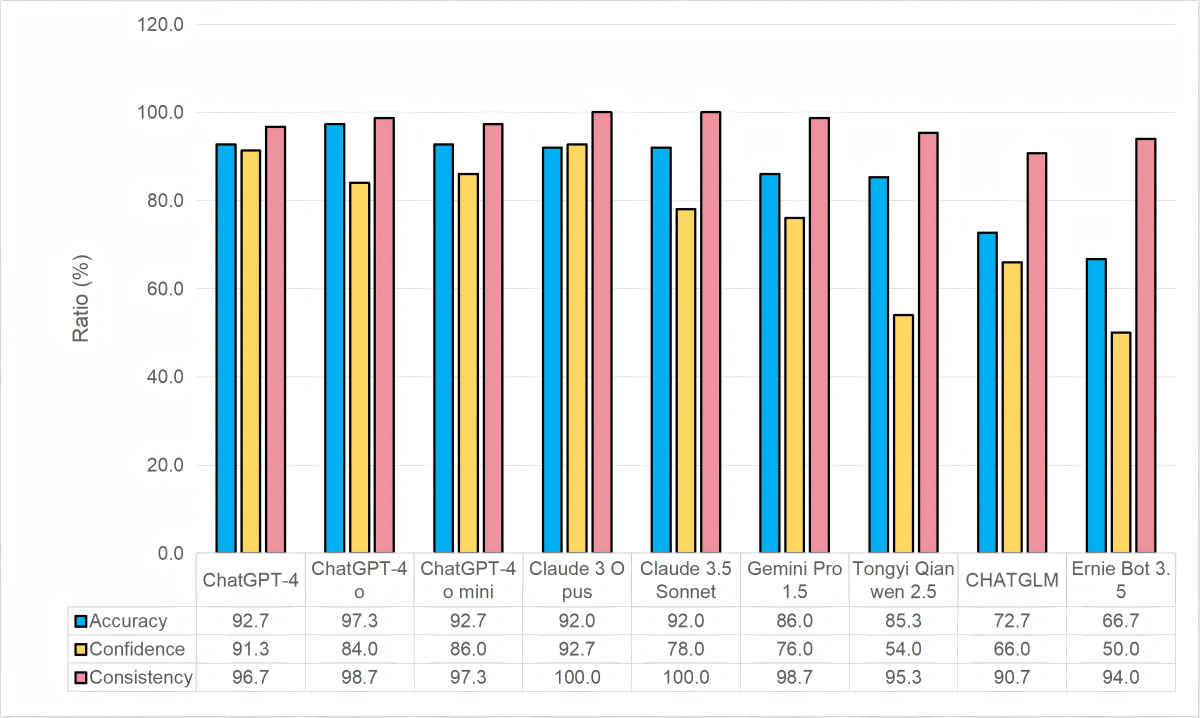
Percentage of accuracy, confidence, and consistency levels of large language models (LLMs) across all 50 questions.

Claude 3 Opus achieved the highest average score of 2.78, followed by ChatGPT-4 with a score of 2.74. The remaining models scored as follows: ChatGPT-4o 2.52, ChatGPT-4o mini 2.58, Claude 3.5, Sonnet 2.34, Gemini 1.5 Pro 2.28, Tongyi Qianwen 2.5 1.62, ChatGLM 1.98, and Ernie Bot 3.5 1.50. Claude 3 Opus and ChatGPT-4 demonstrated significantly higher confidence compared to the other models (Table 1 and Figure 5).

**Table 1:** Confidence and consistency levels of the large language models (LLMs).

Methods	Confidence level	Consistency level
ChatGPT-4	2.74	2.90
ChatGPT-4o	2.52	2.96
ChatGPT-4o mini	2.58	2.92
Claude 3 Opus	2.78	3.00
Claude 3.5 Sonnet	2.34	3.00
Gemini 1.5 Pro	2.28	2.96
Tongyi Qianwen 2.5	1.62	2.86
ChatGLM	1.98	2.72
Ernie Bot 3.5	1.50	2.82

### Consistency

Claude 3 Opus and Claude 3.5 Sonnet achieved the highest score of 3.0, demonstrating their superiority. The remaining models achieved the following consistency scores: ChatGPT-4o: 2.96, Gemini 1.5 Pro: 2.96, ChatGPT-4o mini: 2.92, ChatGPT4: 2.90, Tongyi Qianwen 2.5: 2.86, Ernie Bot 3.5: 2.82, and ChatGLM: 2.71 (Table 1 and Figure 5).

### Performances on Clinical Diagnostics and Treatment Questions

In the comparison of clinical diagnostic problems, the mean accuracy scores are 3 (SD 0), 2.92 (SD 0.39), 2.81 (SD 0.69), 2.81 (0.49), 2.81 (SD 0.63), 2.77 (SD 0.65), 2.77 (SD 0.82), 2.77 (SD 0.82), 2.65 (SD 0.98), 2.38 (SD 1.02), and 2.35 (SD 1.13) for ChatGPT-4o mini, ChatGPT-4o, Tongyi Qianwen 2.5, attending physicians, fellow physicians, ChatGPT-4, Claude 3 Opus, Claude 3.5 Sonnet, Gemini 1.5 Pro, Ernie Bot 3.5, and ChatGLM, respectively. No significant differences were observed between any language modeling group and the various physician groups. Regarding comparisons among physician groups, no significant differences were detected between different physician subgroups (Table 2, Figure 2, and Multimedia Appendices 1 and 4).

**Table 2:** Accuracy score comparisons of large language models (LLMs) with physician groups for clinical diagnostic and treatment questions.

Methods	Value, mean (SD)	FDR^a^-adjusted *P* value		
		Attending physician	Fellow physician	Resident physician
ChatGPT-4	2.77 (0.65)	.84	.97	.66
ChatGPT-4o	2.92 (0.39)	.44	.81	.49
ChatGPT-4o mini	3 (0)	.37	.42	.23
Claude 3 Opus	2.77 (0.82)	.62	.90	.49
Claude 3.5 Sonnet	2.77 (0.82)	.62	.90	.49
Gemini 1.5 Pro	2.65 (0.98)	.84	.97	.66
Tongyi Qianwen 2.5	2.81 (0.69)	.62	.90	.49
ChatGLM	2.35 (1.13)	.44	.44	.49
Ernie Bot 3.5	2.38 (1.02)	.44	.42	.49
Attending physician	2.81 (0.49)	—^b^	.90	.74
Fellow physician	2.81 (0.63)	—	—	.66
Resident physician	2.77 (0.51)	—	—	—

^a^FDR: false discovery rate.

^b^Not applicable.

In Figure 2, 3-star or dot symbols on each violin from top to bottom represent *P* values compared to each LLM to attending, fellow, and resident physicians, using the Wilcoxon rank-sum test. Symbols are as follows: “***” represents *P*<.001, “**” represents .001≤*P*<.01, “*” represents .01≤*P*<.05, and “.” represents *P*≥.05.

### Performances on Radiological Diagnostics Problems

In the comparison of radiological diagnostic problems, the scores were as follows: ChatGPT-4 2.79, ChatGPT-4o 2.92, ChatGPT-4o mini 2.54, Claude 3 Opus 2.75, Claude 3.5 Sonnet 2.75, Gemini 1.5 Pro 2.5, Tongyi Qianwen 2.29, ChatGLM 2.0, and Ernie Bot 3.5 1.58. The attending physicians scored 2.46, the fellow physicians scored 2.04, and the resident physicians scored 2.5. The differences between ChatGPT-4, ChatGPT-4o, ChatGPT-4o mini, Claude 3 Opus, Claude 3.5 Sonnet, and Gemini 1.5 Pro and the fellow physicians were statistically significant (*P*<.05). There were statistically significant differences between ChatGPT-4o and the resident physicians (*P*=.04). In contrast, the differences between Tongyi Qianwen 2.5, ChatGLM, and the physician groups were not statistically significant (*P*>.05). In comparisons among physician groups, the differences were not statistically significant (*P*>.05; Table 3, Multimedia Appendices 1 and 4, and Figure 5).

**Table 3:** Accuracy score comparisons of large language models (LLMs) with physician groups for radiological diagnostic questions.

Methods	Value, mean (SD)	FDR^a^-adjusted *P* value		
		Attending physician	Fellow physician	Resident physician
ChatGPT-4	2.79 (0.66)	.10	<.001	.07
ChatGPT-4o	2.92 (0.41)	.06	<.001	.04
ChatGPT-4o mini	2.54 (0.93)	.51	.01	.45
Claude 3 Opus	2.75 (0.85)	.09	<.001	.07
Claude 3.5 Sonnet	2.75 (0.85)	.09	<.001	.07
Gemini 1.5 Pro	2.5 (1.06)	.50	.01	.41
Tongyi Qianwen 2.5	2.29 (1.08)	.81	.15	.97
ChatGLM	2 (1.1)	.22	.84	.22
Ernie Bot 3.5	1.58 (1.44)	.09	.49	.09
Attending physician	2.46 (0.78)	—^b^	.07	.99
Fellow physician	2.04 (0.75)	—	—	.07
Resident physician	2.5 (0.66)	—	—	—

^a^FDR: false discovery rate.

^b^Not applicable.

In Figure 3, 3-star or dot symbols on each violin from top to bottom represent *P* values compared to each LLM to attending, fellow, and resident physicians, using the Wilcoxon rank-sum test. Symbols are as follows: “***” represents *P*<.001, “**” represents .001≤*P*<.01, “*” represents .01≤*P*<.05, and “.” represents *P*≥.05.

### Performances on Full Set Questions

In the comparison of overall performance, the scores were as follows: ChatGPT-4o 2.92, ChatGPT-4 2.78, ChatGPT-4o mini 2.78, Claude 3 Opus 2.76, Claude 3.5 Sonnet 2.76, Gemini 1.5 Pro 2.58, Tongyi Qianwen 2.56, ChatGLM 2.18, and Ernie Bot 3.5 2.0. The attending physicians scored 2.64, the fellow physicians scored 2.44, and the resident physicians scored 2.64. The differences between ChatGPT-4, ChatGPT-4o, ChatGPT-4o mini, Claude 3 Opus, Claude 3.5 Sonnet, and the fellow physicians were statistically significant (*P*<.05). Significant differences were observed between ChatGPT-4o and resident physicians (*P*=.01) and between ChatGPT-4o and attending physicians (*P*=.03). In the comparisons among physician groups, none of the differences were statistically significant (*P*>.05; Table 4, Multimedia Appendices 1 and 4, and Figure 4).

**Table 4:** Accuracy score comparisons of large language model (LLM) models with physician groups for all 50 questions.

Methods	Value, mean (SD)	FDR^a^-adjusted *P* value		
		Attending physician	Fellow physician	Resident physician
ChatGPT-4	2.78 (0.65)	.13	.006	.09
ChatGPT-4o	2.92 (0.4)	.03	<.001	.01
ChatGPT-4o mini	2.78 (0.68)	.13	.006	.09
Claude 3 Opus	2.76 (0.82)	.08	.003	.05
Claude 3.5 Sonnet	2.76 (0.82)	.08	.003	.05
Gemini 1.5 Pro	2.58 (1.01)	.47	.06	.30
Tongyi Qianwen 2.5	2.56 (0.93)	.84	.18	.67
ChatGLM	2.18 (1.12)	.09	.47	.12
Ernie Bot 3.5	2 (1.29)	.08	.26	.09
Attending physician	2.64 (0.66)	—^b^	.19	.78
Fellow physician	2.44 (0.79)	—	—	.30
Resident physician	2.64 (0.6)	—	—	—

^a^FDR: false discovery rate.

^b^Not applicable.

In Figure 4, 3-star or dot symbols on each violin from top to bottom represented *P* values compared to each LLM model to attending, fellow, and resident physicians, with the Wilcoxon rank-sum test. Symbols are as follows: “***” represents *P*<.001, “**” represents .001≤*P*<.01, “*” represents .01≤*P*<.05, and “.” represents *P*≥.05.

## Discussion

### Principal Findings

With the global population aging, the rising incidence of cancer, and the increasing complexity of treatment options, there is a growing demand for MDT discussions. However, implementing high-level MDTs in underdeveloped countries remains a significant challenge [16], largely because of the knowledge barriers between specialist doctors. An important question is how specialist doctors in less developed regions can achieve the same medical standards as those in developed regions, easily access authoritative and professional medical knowledge, and overcome barriers between specialties. The recently developed chatbot can theoretically provide instant, evidence-based responses, demonstrating significant potential as an ideal tool for enhancing high-quality health care in underserved regions. In our comparison of 9 LLMs, Claude 3 Opus demonstrated the highest confidence score at 2.78, followed by ChatGPT-4 (2.74) and ChatGPT-4o mini (2.58). These scores suggest that these models exhibit a higher degree of certainty when managing complex medical issues. In contrast, Ernie Bot 3.5’s confidence score of 1.50 was significantly lower, indicating a reduced ability to comprehend and process medical problems. These findings underscore the varying levels of proficiency among language models in accurately addressing the same problem. Interestingly, unlike human practitioners, not all chatbots confidently respond to closed-ended questions with a definitive “yes” or “no,” suggesting that training data diversity and model architecture significantly impact diagnostic accuracy. This uncertainty may lead to reduced consistency and accuracy of their outputs, thus limiting their independent use in practical clinical applications, making them inseparable from close collaboration with physicians during decision-making.

In addition, in the consistency assessment, Claude 3 Opus and Claude 3.5 Sonnet achieved the highest scores of 3, followed by ChatGPT-4o and Gemini 1.5 Pro, both scoring 2.96, with ChatGPT-4o mini at 2.92. In contrast, ChatGLM and Ernie Bot 3.5 exhibited lower consistency scores of 2.72 and 2.82, respectively. These results highlight the differences in response stability across the LLMs, a factor that is especially critical for clinical decision support. In clinical applications, particularly in dynamic and complex environments, models lacking sufficient consistency may undermine confidence in their outputs. Although Claude 3 Opus and Claude 3.5 Sonnet surpassed the ChatGPT models in response consistency (scoring 3), the ChatGPT models demonstrated superior performance in diagnostic accuracy. However, none of the models provided correct answers to all question sets. Therefore, while AI introduces new possibilities in fields such as medicine, it also presents challenges that require careful expert scrutiny to avoid imposing additional burdens on patients and health care professionals [17,18].

By comparing the nine LLMs with the responses of nine radiologists on the overall 50 guideline questions, ChatGPT-4o achieved the highest score of 2.92, significantly outperforming all physician groups. The difference between ChatGPT-4o and all physician groups (*P*<.05) was statistically significant. ChatGPT-4, ChatGPT-4o mini, Claude 3 Opus, and Claude 3.5 Sonnet all scored higher on average than the physician groups. However, the difference was statistically significant only for the comparison with fellow physicians (*P*<.05). In contrast, Gemini 1.5 Pro and Tongyi Qianwen 2.5 had lower scores, with no statistically significant differences compared to each group of doctors (*P*>.05). ChatGLM and Ernie Bot 3.5 showed lower scores than all physician groups, but the differences were not statistically significant. No statistically significant differences (*P*>.05) were found in the comparisons between each group of doctors, suggesting that attending physicians, fellow physicians, and resident physicians performed comparably in answering the entire question set. Attending physicians and resident physicians both scored 2.64, which may reflect a more consistent ability to answer certain routine questions across all years of experience. Fellow physicians, on the other hand, scored slightly lower, but the difference was not significant, suggesting that their performance in overall diagnostic and therapeutic tasks remains competitive.

In the evaluation of radiological diagnostic performance, ChatGPT-4o scored 2.92. A significant difference was observed between ChatGPT-4o and resident physicians (*P*=.04), and a significant difference was also detected between ChatGPT-4o and fellow physicians (*P*<.05). ChatGPT-4 achieved a score of 2.79, surpassing all physician groups on average. However, this difference reached statistical significance solely in the comparison with fellow physicians (*P*<.05). The difference between ChatGPT-4o mini and the fellow physicians was statistically significant (*P*=.01). Claude 3 Opus and Claude 3.5 Sonnet both scored 2.75, higher than all physician groups; in particular, the difference between these LLMs and fellow physicians was statistically significant (*P*<.05), suggesting superior performance in radiological diagnostics. Gemini 1.5 Pro scored 2.50, showing small differences compared with attending physicians and resident physicians but significantly higher than fellow physicians (*P*=.01). Tongyi Qianwen 2.5 and ChatGLM scored lower, with no statistically significant differences compared to physician groups (*P*>.05). Ernie Bot 3.5 had the lowest score (1.58) among all LLMs, with no statistical significance compared to any physician group, indicating relatively poorer performance. We conclude that the ChatGPT and Claude series outperform junior medical doctors in addressing complex medical issues, highlighting their potential clinical utility. However, ChatGLM and Ernie Bot 3.5 may require further optimization to improve their performance in medical diagnostics. In the diagnostic radiology domain, the average performance of attending physicians and resident physicians was comparable, whereas the small group of fellow physicians scored lower on average. It is noteworthy that resident physicians, who have recently completed standardized residency training, may be more familiar with the specific guidelines on which the questions were based. Attending physicians, benefiting from extensive clinical experience, would be expected to exhibit an advantage in diagnosing complex imaging findings. The performance variation across the small physician subgroups underscores the preliminary nature of these comparisons.

In the assessment of clinical diagnosis and treatment performance, ChatGPT-4o mini achieved the highest score of 3 among all LLMs, although no statistically significant differences were found when compared with any physician group. ChatGPT-4o scored 2.92 and showed no significant difference compared with physician groups (*P*>.05); however, it outperformed all physician groups on average. Other LLMs, including ChatGPT-4, Claude 3 Opus, and Claude 3.5 Sonnet, exhibited similar performance to physician groups. ChatGLM and Ernie Bot 3.5 showed weaker performance. Overall, the ChatGPT and Claude series of LLMs outperformed some physician groups with lower seniority in complex health care challenges, indicating potential for clinical applications. Further optimization may be necessary for ChatGLM and Ernie Bot 3.5 to enhance their diagnostic performance. Scores between attending physicians and fellow physicians were identical, while resident physicians scored slightly lower; however, these differences did not reach statistical significance. Radiologists of varying experience levels may possess adequate knowledge of clinical guidelines in diagnosis and treatment, particularly when dealing with standardized procedures or common diseases, resulting in minimal differences. Despite differences in experience, attending physicians and fellow physicians demonstrated similar proficiency in applying clinical guidelines.

It is noteworthy that Tongyi Qianwen 2.5 was the only model unable to generate a response to a specific question, instead providing the feedback, “I’m very sorry, but I don’t think I fully understand what you mean. Let’s change the subject first, shall we?” This issue may be attributable to the complexity and specialized nature of the question, or potential biases and gaps in the model’s training data, leading to insufficient knowledge in specific domains. Our data indicate that when Tongyi Qianwen 2.5 fails to respond, the model’s accuracy may be overstated, and its actual performance is significantly below expectations.

### Comparison With Previous Work

In our analysis of the 9 language models, Ernie Bot 3.5, Tongyi Qianwen 2.5, and ChatGLM were more likely to recommend referring the questions to medical experts when faced with specialized imaging terminology and clinical scenarios, rather than providing a definitive answer. In contrast, Claude 3 Opus and the ChatGPT series provided more detailed diagnostic imaging feedback, including recommendations for additional biopsies to support diagnosis and an emphasis on multidisciplinary collaboration. Regarding clinical considerations for surgery, the ChatGPT series excelled by offering surgical recommendations for breast cancer patients, while also demonstrating conventional surgical approaches and discussing recent therapeutic advancements. Furthermore, the ChatGPT family outperformed other language models in breast cancer diagnosis and treatment, with each model excelling in its respective area of expertise. This finding highlights the critical need to select the most appropriate language model based on task-specific performance, thereby enhancing problem-solving across various scenarios. These interactive language models provided feedback significantly faster than breast cancer radiologists, reducing time costs and increasing productivity. With continued iteration and improvement, ChatGPT has demonstrated considerable potential in radiology, including concise report generation, support for medical education, clinical decision aids, patient communication optimization, and data analysis [19,20]. Analyzing the reasons for comparable performance between leading language models reveals several key advantages: (1) large-scale, high-quality training data: both the developers of Claude 3 Opus and Chat GPT have invested substantial resources in acquiring and curating authoritative medical literature, textbooks, and specialized professional databases; (2) comparable model scale and architecture: as state-of-the-art LLMs, both likely use similar parameter scales and analogous underlying architectural designs; (3) equivalent alignment methodologies: both organizations have likely implemented similar reinforcement learning from human feedback techniques, using extensive human evaluator input to enhance the accuracy of model outputs; (4) competition-driven enhancement: market competition has compelled both companies to continuously improve model performance, particularly in high-value vertical domains such as health care. This competitive environment has resulted in both products demonstrating enhanced logical reasoning capabilities; (5) domain-specific knowledge processing: both models have likely incorporated specialized training methodologies for processing technical terminology and complex semantic relationships within specialized conceptual frameworks; (6) parallel safety control mechanisms: in high-risk domains such as medicine, both models have presumably implemented stringent output controls and prudence measures to ensure responsible performance. Given the continuous evolution of LLMs, their capabilities in complex domains, particularly medicine, are rapidly advancing. Future research should further investigate the potential of these models to maximize their value in clinical diagnosis and treatment [21].

However, LLMs were not specifically trained for the medical field and rely on large datasets collected from public sources, such as web pages, books, and websites. Chinese LLMs have reported average accuracy in general medical question-answering of around 82%; yet, performance drops significantly in complex clinical reasoning tasks, such as breast cancer diagnosis (below 50%) [22]. Advanced models, such as ChatGPT-3.5, ChatGPT-4.0, and Claude-2, achieve slightly higher performance (approximately 60%) in breast cancer clinical assessments, though this still highlights critical limitations in high-stakes medical applications [23]. Despite the vast amount of training data, these models lack comprehensive common sense, which limits their reasoning ability. The literature suggests that LLM performance can be significantly enhanced by guiding reasoning steps incrementally, particularly in tasks involving longer reasoning chains [24,25]. In our study, however, the 9 LLMs were unable to accurately diagnose all rigorous and logical medical problems, and a “curse of reversal” phenomenon was observed. Furthermore, unlike real-time search engines such as Google and Bing, LLMs can only generate responses based on their training data and do not have real-time access to current information [26,27]. In addition, LLMs cannot reliably differentiate between factual and fictional statements, posing a potential risk as sources of misinformation in certain cases [28-30]. Moreover, the training data for these models are not publicly available, and the sources of their information are not disclosed, making it difficult to verify the accuracy of their outputs [31]. Therefore, radiologists must continue to rely on traditional evidence-based education and approach LLMs critically, cross-referencing their outputs with credible medical sources [32].

### Limitations

This study has several limitations. First, our findings are subject to the inherent limitations of the author-crafted, closed-ended question format. Although designed for objective assessment, this format may not fully capture the reasoning capabilities of LLMs and could potentially underestimate their performance in real-world, open-ended clinical scenarios. Second, the binary definition of a “confident” response as a direct “yes” or “no” may not adequately reflect clinical decision-making, where acknowledging uncertainty is a sign of expertise. This could have introduced bias by favoring LLMs that generate definitive answers over physicians who appropriately consider diagnostic nuances. Third, the physician sample size, while sufficient for initial comparisons, remains relatively small and may not be fully representative of the broader population of radiologists, which could affect the generalizability of our pairwise comparisons. Fourth, the study’s generalizability is constrained by its specific design: the assessment is limited to 50 selected questions that do not encompass all aspects of the guidelines, and the performance of the LLMs was evaluated on a single, specific task (answering structured concordance questions). Consequently, the results may not be directly extrapolated to other clinical datasets or different natural language processing tasks. Fifth, the study conditions, including a strict 30-minute time limit and a “no-consultation” rule for the participating physicians, may not reflect real-world clinical practice, where complex cases often benefit from more time and collaborative discussion. These methodological choices, while necessary for standardization, may have disadvantaged the physicians and thus the conclusions of this study must be heavily qualified by this context. In addition, it has been reported that LLMs may generate different responses to identical prompts at different times, due to model updates [31,33]. Future studies should use larger and more diverse physician cohorts, incorporate more nuanced assessments of confidence, explore the impact of different time constraints and collaborative settings, and use a broader range of question types and clinical tasks to validate and extend our findings.

### Future Directions

In medical AI applications, LLMs participating in clinical decision-making face critical responsibility challenges, including decision output liability, misinformation risks, and privacy breach accountability [30]. Typically, LLMs do not independently generate decision outputs but function as tools for physicians. Clinicians must integrate guidelines, relevant literature, and their clinical expertise to formulate final clinical decisions. When using LLMs, it is essential to conceal patients’ identifying information to safeguard privacy.

This necessitates establishing explicit legal frameworks that delineate responsibility boundaries among developers, health care institutions, and physicians, while constructing comprehensive accountability mechanisms [34,35]. Concurrently, LLMs present a risk of generating inaccurate medical information that could potentially mislead diagnostic and therapeutic decisions. To address this concern, model developers must implement rigorous content review protocols and ensure professional, authoritative verification of training corpora.

### Conclusions

In conclusion, within the context of our study, Claude 3 Opus and the ChatGPT series, particularly ChatGPT-4o, excelled in addressing breast cancer–related guideline questions and demonstrated significant potential for clinical application. In contrast, the less consistent performance of other LLMs, especially Ernie Bot 3.5 and ChatGLM, suggests that both confidence and consistency should be carefully considered when selecting and applying LLMs to ensure efficacy and stability in clinical practice. This preliminary evaluation indicates that LLMs can provide radiologists with extensive cross-disciplinary knowledge, potentially enhancing contributions within MDTs. However, they cannot fully replace human expertise, particularly in complex diagnostic scenarios that require nuanced decision-making honed by clinical experience.

## Data Availability

The datasets generated or analyzed during this study are available from the corresponding author upon reasonable request.

## Appendix

Multimedia Appendix 1 Scores of large language models (LLMs) and physician groups on 50 questions, with comparison analysis.

Multimedia Appendix 2 Comparison of accuracy scores between large language models (LLMs) and physicians across 2 domains.

Multimedia Appendix 3 Scores of large language models (LLMs) and physician groups on 50 questions, including raw *P* values for comparison.

Multimedia Appendix 4 Specific responses from 9 large language models (LLMs) to the 50 questions (each question asked 3 times) and the corresponding physicians’ responses.

## Abbreviations

AI: artificial intelligence
ACR BI-RADS: American College of Radiology Breast Imaging-Reporting and Data System
LLM: large language model
MDT: multidisciplinary team
NCCN: National Comprehensive Cancer Network
