# Correction: Evaluation of Large Language Models for Radiologists' Support in Multidisciplinary Breast Cancer Teams: Comparative Study

**DOI:** 10.2196/97580

**Published:** 2026-05-07

**Authors:** Hong Jiang, Chun Yang, Wenbin Zhou, Cheng-liang Yin, Shan Zhou, Rui He, Guanghui Ran, Wujie Wang, Meixian Wu, Juan Yu

**Affiliations:** 1 Faculty of Medicine, Macau University of Science and Technology Macao China; 2 Department of Statistics, Zhuhai Clinical Medical College of Jinan University Zhuhai, GuangDong China; 3 Qiandongnan Prefecture Hospital of Traditional Chinese Medicine Kai Li China; 4 Guangdong Provincial Key Laboratory of Tumor Interventional Diagnosis and Treatment Zhuhai China; 5 Department of Medical Innovation Research, Chinese PLA General Hospital Beijing China; 6 Cancer Virology Program, UPMC Hillman Cancer Center, University of Pittsburgh School of Medicine Pittsburgh, PA United States; 7 Department of Microbiology and Molecular Genetics, University of Pittsburgh School of Medicine Pittsburgh, PA United States; 8 Grammar and Cognition Lab (GraC), Department of Translation & Language Sciences, Universitat Pompeu Fabra Barcelona Spain; 9 Department of Radiology Shenzhen Second People's Hospital The First Affiliated Hospital of Shenzhen University Shenzhen China

The incorrectly listed grant number “112175605105” has been replaced with the formal grant number “2023A0505050118,” which was supported by the Department of Science and Technology of Guangdong Province. All other funding details remain unchanged. This correction does not affect the scientific findings, data, or conclusions of the study.

The correction will appear in the online version of the paper on the JMIR Publications website, together with the publication of this correction notice. Because this was made after submission to PubMed, PubMed Central, and other full-text repositories, the corrected article has also been resubmitted to those repositories.
