# Supplementary material for: Evaluation of Large Language Models for Radiologists’ Support in Multidisciplinary Breast Cancer Teams: Comparative Study
Source: JMIR Med Inform. 2026 Feb 2;14:e68182. doi: 10.2196/68182 (PMC12910264; doi:10.2196/68182)
Supplement: Multimedia Appendix 2 [file medinform_v14i1e68182_app2.docx]

Comparison of accuracy scores between large language models (LLMs) and physicians across 2 domains.

|  | | | | | | | | | Radiological  diagnosis | | | Clinical diagnosis and treatment | | | Total | | |  |  |
| --- | --- | --- | --- | --- | --- | --- | --- | --- | --- | --- | --- | --- | --- | --- | --- | --- | --- | --- | --- |
| No of questions | | | | | | | | | 24 | | | 26 | | | 50 | | |  |  |
| **ChatGPT-4** (**1), n (%)** | | | | | | | | | | | | | | | | | |  |  |
|  | | | | | Incorrect | | | | 2(8.33) | | | 3(11.54) | | | 5(10.00) | | |  |  |
|  | | | | | Correct | | | | 22(91.67) | | | 23(88.46) | | | 45(90.00) | | |  |  |
| **ChatGPT-4** (**2), n (%)** | | | | | | | | | | | | | | | | | |  |  |
|  | | | | | Incorrect | | | | 1(4.17) | | | 1(3.85) | | | 2(4.00) | | |  |  |
|  | | | | | Correct | | | | 23(95.83) | | | 25(96.15) | | | 48(96.00) | | |  |  |
| **ChatGPT-4 (3)** **, n (%)** | | | | | | | | | | | | | | | | | |  |  |
|  | | | | | Incorrect | | | | 2(8.33) | | | 2(7.69) | | | 4(8.00) | | |  |  |
|  | | | | | Correct | | | | 22(91.67) | | | 24(92.31) | | | 46(92.00) | | |  |  |
| **ChatGPT-4o（1）,n(%)** | | | | | | | | | | | | | | | | | |  |  |
|  | | | | Incorrect | | | | | 0(0.00) | | | 1(3.85) | | | 1(2.00) | | |  |  |
|  | | | | Correct | | | | | 24(100.00) | | | 25(96.15) | | | 49(98.00) | | |  |  |
| **ChatGPT-4o（2）,n(%)** | | | | | | | | | | | | | | | | | |  |  |
|  | Incorrect | | | | | | | | 1(4.17) | | | 1(3.85) | | | 2(4.00) | | |  |  |
|  | Correct | | | | | | | | 23(95.83) | | | 25(96.15) | | | 48(96.00) | | |  |  |
| **ChatGPT-4o（3）,n(%)** | | | | | | | | | | | | | | | | | |  |  |
|  | | | | | | | Incorrect | | 1(4.17) | | | 0(0.00) | | | 1(2.00) | | |  |  |
|  | | | | | | | Correct | | 23(95.83) | | | 26(100.00) | | | 49(98.00) | | |  |  |
| **ChatGPT-4o mini（1）,n(%)** | | | | | | | | | | | | | | | | | |  |  |
|  | | | | | | | | Incorrect | 3(12.50) | | | 0(0.00) | | | 3(6.00) | | |  |  |
|  | | | | | | | | Correct | 21(87.50) | | | 26(100.00) | | | 47(94.00) | | |  |  |
| **ChatGPT-4o mini（2）,n(%)** | | | | | | | | | | | | | | | | | |  |  |
|  | | | | | | | Incorrect | | 5(20.83) | | | 0(0.00) | | | 5(10.00) | | |  |  |
|  | | | | | | | Correct | | 19(79.17) | | | 26(100.00) | | | 45(90.00) | | |  |  |
| **ChatGPT-4o mini（3）,n(%)** | | | | | | | | | | | | | | | | | |  |  |
|  | | | | Incorrect | | | | | 3(12.50) | | | 0(0.00) | | | 3(6.00) | | |  |  |
|  | | | | Correct | | | | | 21(87.50) | | | 26(100.00) | | | 47(94.00) | | |  |  |
| **Claude 3 Opus （1）,n(%)** | | | | | | | | | | | | | | | | | |  |  |
|  | | | | | Incorrect | | | | 2(8.33) | | | 2(7.69) | | | 4(8.00) | | |  |  |
|  | | | | | Correct | | | | 22(91.67) | | | 24(92.31) | | | 46(92.00) | | |  |  |
| **Claude 3 Opus （2）,n(%)** | | | | | | | | | | | | | | | | | |  |  |
|  | | | Incorrect | | | | | | 2(8.33) | | | 2(7.69) | | | 4(8.00) | | |  |  |
|  | | | Correct | | | | | | 22(91.67) | | | 24(92.31) | | | 46(92.00) | | |  |  |
| **Claude 3 Opus （3）,n(%)** | | | | | | | | | | | | | | | | | |  |  |
|  | | | Incorrect | | | | | | 2(8.33) | | | 2(7.69) | | | 4(8.00) | | |  |  |
|  | | | Correct | | | | | | 22(91.67) | | | 24(92.31) | | | 46(92.00) | | |  |  |
| **Claude 3.5 Sonnet（1）,n(%)** | | | | | | | | | | | | | | | | | |  |  |
|  | Incorrect | | | | | | | | 2(8.33) | | | 2(7.69) | | | 4(8.00) | | |  |  |
|  | Correct | | | | | | | | 22(91.67) | | | 24(92.31) | | | 46(92.00) | | |  |  |
| **Claude 3.5 Sonnet（2）,n(%)** | | | | | | | | | | | | | | | | | |  |  |
|  | Incorrect | | | | | | | | 2(8.33) | | | 2(7.69) | | | 4(8.00) | | |  |  |
|  | Correct | | | | | | | | 22(91.67) | | | 24(92.31) | | | 46(92.00) | | |  |  |
| **Claude 3.5 Sonnet（3）,n(%)** | | | | | | | | | | | | | | | | | |  |  |
|  | | Incorrect | | | | | | | 2(8.33) | | | 2(7.69) | | | 4(8.00) | | |  |  |
|  | | Correct | | | | | | | 22(91.67) | | | 24(92.31) | | | 46(92.00) | | |  |  |
| **Gemini 1.5 Pro （1）,n(%)** | | | | | | | | | | | | | | | | | |  |  |
|  | | | | | Incorrect | | | | 3(12.50) | | | 3(11.54) | | | 6(12.00) | | |  |  |
|  | | | | | Correct | | | | 21(87.50) | | | 23(88.46) | | | 44(88.00) | | |  |  |
| **Gemini 1.5 Pro （2）,n(%)** | | | | | | | | | | | | | | | | | |  |  |
|  | Incorrect | | | | | | | | 4(16.67) | | | 3(11.54) | | | 7(14.00) | | | |  |
|  | Correct | | | | | | | | 20(83.33) | | | 23(88.46) | | | 43(86.00) | | |  |  |
| **Gemini 1.5 Pro （3）,n(%)** | | | | | | | | | | | | | | | | | |  |  |
|  | | | | | Incorrect | | | | 5(20.83) | | | 3(11.54) | | | 8(16.00) | | |  |  |
|  | | | | | Correct | | | | 19(79.17) | | | 23(88.46) | | | 42(84.00) | | |  |  |
| **Tongyi Qianwen 2.5 （1）,n(%)** | | | | | | | | | | | | | | | | | |  |  |
|  | | | | | | | | Incorrect | 8(33.33) | | | 1(3.85) | | | 9(18.00) | | |  |  |
|  | | | | | | | | Correct | 16(66.67) | | | 25(96.15) | | | 41(82.00) | | |  |  |
| **Tongyi Qianwen 2.5 （2）,n(%)** | | | | | | | | | | | | | | | | | |  |  |
|  | | | | | | | | Incorrect | 4(16.67) | | | 2(7.69) | | | 6(12.00) | | |  |  |
|  | | | | | | | Correct | | 20(83.33) | | | 24(92.31) | | | 44(88.00) | | |  |  |
| **Tongyi Qianwen 2.5 （3）,n(%)** | | | | | | | | | | | | | | | | | |  |  |
|  | | Incorrect | | | | | | | 5(20.83) | | | 2(7.69) | | | 7(14.00) | | |  |  |
|  | | Correct | | | | | | | 19(79.17) | | | 24(92.31) | | | 43(86.00) | | |  |  |
| **ChatGLM （1）,n(%)** | | | | | | | | | | | | | | | | | |  |  |
|  | | Incorrect | | | | | | | 13(54.17) | | | 3(11.54) | | | 16(32.00) | | |  |  |
|  | | Correct | | | | | | | 11(45.83) | | | 23(88.46) | | | 34(68.00) | | |  |  |
| **ChatGLM （2）,n(%)** | | | | | | | | | | | | | | | | | |  |  |
|  | Incorrect | | | | | | | | 7(29.17) | | | 7(26.92) | | | 14(28.00) | | | |  |
|  | Correct | | | | | | | | 17(70.83) | | | 19(73.08) | | | 36(72.00) | | |  |  |
| **ChatGLM （3）,n(%)** | | | | | | | | | | | | | | | | | |  |  |
|  | | | | | | Incorrect | | | 4(16.67) | | | 7(26.92) | | | 11(22.00) | | |  |  |
|  | | | | | | Correct | | | 20(83.33) | | | 19(73.08) | | | 39(78.00) | | |  |  |
| **Ernie Bot3.5 （1）,n(%)** | | | | | | | | | | | | | | | | | |  |  |
|  | | | | | | Incorrect | | | 12(50.00) | | | 7(26.92) | | | 19(38.00) | | |  |  |
|  | | | | | | Correct | | | 12(50.00) | | | 19(73.08) | | | 31(62.00) | | |  |  |
| **Ernie Bot3.5 （2）,n(%)** | | | | | | | | | | | | | | | | | |  |  |
|  | | | | | | Incorrect | | | 11(45.83) | | | 5(19.23) | | | 16(32.00) | | |  |  |
|  | | | | | | Correct | | | 13(54.17) | | | 21(80.77) | | | 34(68.00) | | |  |  |
| **Ernie Bot3.5 （3）,n(%)** | | | | | | | | | | | | | | | | | |  |  |
|  | Incorrect | | | | | | | | 11(45.83) | | | 4(15.38) | | | 15(30.00) | | | |  |
|  | Correct | | | | | | | | 13(54.17) | | | 22(84.62) | | | 35(70.00) | | |  |  |
| **Attending physician（1）,n(%)** | | | | | | | | | | | | | | | | | |  |  |
|  | | | | | Incorrect | | | | 4(16.67) | | | 1(3.85) | | | 5(10.00) | | |  |  |
|  | | | | | Correct | | | | 20(83.33) | | | 25(96.15) | | | 45(90.00) | | |  |  |
| **Attending physicians（2）,n(%)** | | | | | | | | | | | | | | | | | |  |  |
|  | Incorrect | | | | | | | | 6(25.00) | | | 2(7.69) | | | 8(16.00) | | |  |  |
|  | Correct | | | | | | | | 18(75.00) | | | 24(92.31) | | | 42(84.00) | | |  |  |
| **Attending physician（3）,n(%)** | | | | | | | | | | | | | | | | | |  |  |
|  | | | | | Incorrect | | | | 3(12.50) | | | 2(7.69) | | | 5(10.00) | | |  |  |
|  | | | | | Correct | | | | 21(87.50) | | | 24(92.31) | | | 45(90.00) | | |  |  |
| **Fellow physician（1）,n(%)** | | | | | | | | | | | | | | | | | |  |  |
|  | Incorrect | | | | | | | | | 4(16.67) | | | 1(3.85) | | | 5(10.00) | | |  |
|  | Correct | | | | | | | | 20(83.33) | | | 25(96.15) | | | 45(90.00) | | |  |  |
| **Fellow physician（2）,n(%)** | | | | | | | | | | | | | | | | | |  |  |
|  | Incorrect | | | | | | | | | 14(58.33) | | | 3(11.54) | | | 17(34.00) | | |  |
|  | Correct | | | | | | | | 10(41.67) | | | 23(88.46) | | | 33(66.00) | | |  |  |
| **Fellow physician（3）,n(%)** | | | | | | | | | | | | | | | | | |  |  |
|  | | | | | | | Incorrect | | 5(20.83) | | | 1(3.85) | | | 6(12.00) | | |  |  |
|  | | | | | | | Correct | | 19(79.17) | | | 25(96.15) | | | 44(88.00) | | |  |  |
| **Resident physician（1）,n(%)** | | | | | | | | | | | | | | | | | |  |  |
|  | Incorrect | | | | | | | | | | 7(29.17) | | | 2(7.69) | | | 9(18.00) | | |
|  | Correct | | | | | | | | | | 17(70.83) | | | 24(92.31) | | | 41(82.00) | | |
| **Resident physician（2）,n(%)** | | | | | | | | | | | | | | | | | |  |  |
|  | | | | | | | Incorrect | | 2(8.33) | | | 2(7.69) | | | 4(8.00) | | |  |  |
|  | | | | | | | Correct | | 22(91.67) | | | 24(92.31) | | | 46(92.00) | | |  |  |
| **Resident physician（3）,n(%)** | | | | | | | | | | | | | | | | | |  |  |
|  | | | | | Incorrect | | | | 3(12.50) | | | 2(7.69) | | | 5(10.00) | | |  |  |
|  | | | | | Correct | | | | 21(87.50) | | | 24(92.31) | | | 45(90.00) | | |  |  |
