# Supplementary material for: Evaluation of Large Language Models for Radiologists’ Support in Multidisciplinary Breast Cancer Teams: Comparative Study
Source: JMIR Med Inform. 2026 Feb 2;14:e68182. doi: 10.2196/68182 (PMC12910264; doi:10.2196/68182)
Supplement: Multimedia Appendix 3 [file medinform_v14i1e68182_app3.docx]

| Methods | Mean | *P* value | | |
| --- | --- | --- | --- | --- |
|  |  | Attending physician | Fellow physician | Resident physician |
| ChatGPT-4 | 2.77 | .81 | .97 | .57 |
| ChatGPT-4o | 2.92 | .19 | .32 | .10 |
| ChatGPT-4o mini | 3.00 | .04 | .08 | .02 |
| Claude 3 Opus | 2.77 | .48 | .71 | .30 |
| Claude 3.5 Sonnet | 2.77 | .48 | .71 | .30 |
| Gemini 1.5 Pro | 2.65 | .84 | .93 | .60 |
| Tongyi Qianwen 2.5 | 2.81 | .47 | .69 | .29 |
| ChatGLM | 2.35 | .20 | .13 | .31 |
| Ernie Bot 3.5 | 2.38 | .13 | .08 | .22 |
| Attending physician | 2.81 | \ | .72 | .74 |
| Fellow physician | 2.81 | \ | \ | .48 |
| Resident physician | 2.77 | \ | \ | \ |

**Table S1.** Accuracy score comparisons of LLM models with physician groups regarding clinical diagnostic and treatment questions.

| Methods | Mean | *P* value | | |
| --- | --- | --- | --- | --- |
|  |  | Attending physician | Fellow physician | Resident physician |
| ChatGPT-4 | 2.79 | .05 | <.001 | .03 |
| ChatGPT-4o | 2.92 | .007 | <.001 | .004 |
| ChatGPT-4o mini | 2.54 | .45 | .008 | .37 |
| Claude 3 Opus | 2.75 | .04 | <.001 | .02 |
| Claude 3.5 Sonnet | 2.75 | .04 | <.001 | .02 |
| Gemini 1.5 Pro | 2.50 | .39 | .009 | .30 |
| Tongyi Qianwen 2.5 | 2.29 | .81 | .12 | .88 |
| ChatGLM | 2.00 | .15 | .84 | .14 |
| Ernie Bot 3.5 | 1.58 | .04 | .44 | .05 |
| Attending physician | 2.46 | \ | .05 | .99 |
| Fellow physician | 2.04 | \ | \ | .03 |
| Resident physician | 2.50 | \ | \ | \ |

**Table S2.** Accuracy score comparisons of LLM models with physician groups regarding radiological diagnostic questions.

| Methods | Mean | *P* value | | |
| --- | --- | --- | --- | --- |
|  |  | Attending physician | Fellow physician | Resident physician |
| ChatGPT-4 | 2.78 | .10 | .003 | .05 |
| ChatGPT-4o | 2.92 | .003 | <.001 | .001 |
| ChatGPT-4o mini | 2.78 | .10 | .003 | .04 |
| Claude 3 Opus | 2.76 | .04 | .001 | .01 |
| Claude 3.5 Sonnet | 2.76 | .04 | .001 | .01 |
| Gemini 1.5 Pro | 2.58 | .42 | .04 | .24 |
| Tongyi Qianwen 2.5 | 2.56 | .84 | .12 | .61 |
| ChatGLM | 2.18 | .05 | .47 | .08 |
| Ernie Bot 3.5 | 2.00 | .02 | .23 | .03 |
| Attending physician | 2.64 | \ | .15 | .78 |
| Fellow physician | 2.44 | \ | \ | .22 |
| Resident physician | 2.64 | \ | \ | \ |

**Table S3.** Accuracy score comparisons of LLM models with physician groups regarding all 50 questions.
